# Effect of Parenchymal Stiffness on Canine Airway Size with Lung Inflation

**DOI:** 10.1371/journal.pone.0010332

**Published:** 2010-04-26

**Authors:** Robert H. Brown, David W. Kaczka, Wayne Mitzner

**Affiliations:** 1 Department of Anesthesiology and Critical Care Medicine, Johns Hopkins University, Baltimore, Maryland, United States of America; 2 Department of Environmental Health Sciences, Johns Hopkins University, Baltimore, Maryland, United States of America; 3 Department of Radiology, Johns Hopkins University, Baltimore, Maryland, United States of America; 4 Department of Biomedical Engineering, Johns Hopkins University, Baltimore, Maryland, United States of America; Johns Hopkins School of Medicine, United States of America

## Abstract

Although airway patency is partially maintained by parenchymal tethering, this structural support is often ignored in many discussions of asthma. However, agonists that induce smooth muscle contraction also stiffen the parenchyma, so such parenchymal stiffening may serve as a defense mechanism to prevent airway narrowing or closure. To quantify this effect, specifically how changes in parenchymal stiffness alter airway size at different levels of lung inflation, in the present study, we devised a method to separate the effect of parenchymal stiffening from that of direct airway narrowing. Six anesthetized dogs were studied under four conditions: baseline, after whole lung aerosol histamine challenge, after local airway histamine challenge, and after complete relaxation of the airways. In each of these conditions, we used High resolution Computed Tomography to measure airway size and lung volume at five different airway pressures (0, 12, 25, 32, and 45 cm H_2_O). Parenchymal stiffening had a protective effect on airway narrowing, a fact that may be important in the airway response to deep inspiration in asthma. When the parenchyma was stiffened by whole lung aerosol histamine challenge, at every lung volume above FRC, the airways were larger than when they were directly challenged with histamine to the same initial constriction. These results show for the first time that a stiff parenchyma per se minimizes the airway narrowing that occurs with histamine challenge at any lung volume. Thus in clinical asthma, it is not simply increased airway smooth muscle contraction, but perhaps a lack of homogeneous parenchymal stiffening that contributes to the symptomatic airway hyperresponsiveness.

## Introduction

It has been suggested that the loss of protection normally offered by a deep inspiration against augmented airway narrowing in asthmatic subjects reflects an intrinsic pathophysiology of this disease [1], [2], [3], [4], [5]. Although it seems clear that properties of airway smooth muscle must be involved in this functional difference, the properties of the parenchyma that surround the airways also may play an important role. Parenchymal tethering produces a transmural load on the airways that depends on both the distensibility of the lung (bulk elastic modulus) and its ability to deform (shear modulus) [28]. With lung inflation, both of these variables increase. Thus, if the parenchyma is stiffened, for a given level of lung inflation, the distending forces on the airways will be increased relative to the baseline state. There is good evidence that parenchymal stiffness is increased with agonists that cause smooth muscle contraction, and several investigations have emphasized the potential importance of the parenchyma in asthma [6], [7], [8]. Experimental studies in animal models and human studies clearly show that methacholine can stiffen the lung parenchyma, increasing both elastance and tissue resistance [9], [10], and these changes will affect the airways under both static and dynamic conditions. They also suggest that the abnormal response in asthma to a deep inspiration may be related to the interaction between airway smooth muscle and its stiffened parenchymal environment. This stiffer lung surrounding the airway may also serve as a protective response during an asthma attack by limiting airway narrowing or closure, but this hypothesis has not been evaluated. One reason for this lack of knowledge is that the ability to directly measure and separate the effect of parenchymal stiffening on the airway caliber has not been available. In the present study, we have designed an in vivo protocol that allows separation of the effect of airway contraction with and without stiffened parenchyma at different lung volumes. This protocol compares a whole lung aerosol challenge that contracts airways and stiffens the parenchyma with local challenges via a bronchoscopically-guided catheter that contracts only individual airways. Results show for the first time that a stiff parenchyma per se minimizes the airway narrowing that occurs with airway smooth muscle constriction at any lung volume.

## Methods

The study protocol was approved and signed by The Johns Hopkins Animal Care and Use Committee, with the officially approved protocol #DO08H03. All handling of the animals from anesthesia to recovery were done in strict accordance with the guidelines presented in both the Public Health Service Policy on Humane Care and Use of Laboratory Animals (Office of Laboratory Animal Welfare, National Institutes of Health, Bethesda, MD) and the Guide for the Care and Use of Laboratory Animals (Institute of Laboratory Animal Resources Commission on Life Sciences, National Research Council, Washington, DC). Six dogs weighing approximately 20 kg were anesthetized with pharmaceutical grade thiopental sodium (15 mg/kg induction dose followed by 10 mg/kg/hr intravenous maintenance dose). A stable depth of anesthesia was monitored by lash reflex, heart rate, and respiration, and airway pressure and end tidal CO_2_ are measured and used to assess the adequacy of ventilation. After induction of anesthesia, the dogs were paralyzed during the imaging with 0.5 mg/kg of succinylcholine to ensure no respiratory motion. Following endotracheal intubation with an 8.0 mm ID endotracheal tube, the dogs were placed supine and their lungs were ventilated with room air using a volume-cycled ventilator (Harvard Apparatus, Millus, MA) at a tidal volume of 15 ml/kg and a rate of 18 breaths/minute. In four of the dogs, we inserted a thin-walled latex esophageal balloon inflated with 0.8 ml of air which was positioned in the lower third of the esophagus to estimate pleural pressure changes [11]. Transpulmonary pressure (Ptp) was measured as the difference between the airway pressure measured at the end of the endotracheal tube and the esophageal pressure. Following the experimental procedures all animals were kept under direct observation until they were breathing normally. The investigators and/or a trained technician remained as long as necessary to ensure that no animal received less than adequate monitoring until they had fully recovered from the anesthesia and exhibit normal behavior.

### Protocol

Each dog served as its own control. The dogs were anesthetized and ventilated as described above. They were studied under four conditions on 2 separate days at least 2 weeks apart, in random order. The four conditions were studied on two separate days. On one of the days we measured the following: 1) baseline diameter and volume with no exogenous challenge; and followed by 2) measurements after total lung challenge with aerosolized histamine. On the other day we measured: 3) the lungs and airways after relaxation with atropine; and then followed by 4) measurements after a local histamine challenge to 5 or 6 selected airway segments of less than 1 cm in length [12]. To standardize lung volume history, prior to the first High Resolution Computed Tomography (HRCT) scan series, the airway pressure was increased to 45 cmH_2_O, held for 5 seconds and then released, followed by normal ventilation.

On one experimental day, baseline HRCT scans were acquired (details below), followed by an aerosol challenge of histamine (200 mg/ml, 5 breaths to 15 cm H_2_O, Sigma Chemical, St Louis MO). The aerosol challenges were administered by a Hudson 3000 nebulizer (Hudson, Temecula, CA) driven by compressed oxygen at 10 L/min. Under test conditions with an operating pressure of 50 psi and a flow rate of 10 L/min, the nebulizer produced particles of mass median diameter of 3.1 µm with a geometric SD of 3.2. Given such a relatively dispersed particle size distribution, good central and peripheral distribution should have been achieved [13]. Approximately 1 mL of solution was delivered by the nebulizer during this whole lung challenge. Three minutes later, the HRCT scans were acquired.

On the second experimental day, intravenous atropine (0.2 mg/kg) was administered, a dose previously shown to completely block vagal tone in the dog [14]. After 10 minutes, relaxed airway scans were acquired. A second method of airway constriction was done by local atomization of agonist delivered directly to the epithelium of the same airway locations that were measured during the aerosol challenge. The atomization was accomplished with a specially designed catheter that could be placed with bronchoscopic visualization [12]. This catheter consisted of PE-190 tubing with a short (2-mm) plastic tube inserted into the tip. This tube had six tiny (0.15-mm) side holes drilled circumferentially 1 mm from the end, and the end was plugged with a 1-mm stainless steel rod. This metal plug aided visualization in the computed tomography (CT) scanner. In practice, the catheter was filled outside the lung with the desired agonist concentration and advanced 1.5 cm beyond the tip of the bronchoscope. Rapid injections of 20 µL boluses caused the liquid to be sprayed radially on the adjacent airway wall, with minimal axial spread [12]. Five to six airways were identified in each dog and challenged with histamine (20 µL per bolus per airway, concentration: 200 mg/ml, Sigma Chemical, St Louis MO). The histamine dose was chosen based on pilot studies on nonexperimental days. It took between 15–20 seconds to challenge the 5 or 6 airways. All airway locations to be challenged were identified prior to the deep inspiration to set volume history. For the local challenges, the ventilator was stopped, a single dose was administered to each of the 5 or 6 airway locations over 15–20 seconds, the bronchoscope was removed, and normal ventilation was resumed. After 3 minutes, ventilation was stopped for the HRCT scan acquisition. For each condition on both experimental days, the airway sizes and lung volumes were measured at 5 airway pressures in increasing order: 0, 12, 25 32, and 45 cm H_2_O. Each scan series took approximately 8 seconds to complete. The dogs were ventilated normally between scanning.

### Imaging and Analysis

HRCT scans were obtained with a Sensation-16 scanner (Siemens, Iselin, NJ) using a spiral mode to acquire approximately 300 CT images during an 8 second breath hold (apnea) at 137 kVp, and 165 mA. The total lung volumes (apex to base) were scanned. The images were reconstructed at 1 mm slice thickness with a 512x512 matrix using a 230 mm field of view and a high spatial frequency (resolution) algorithm that enhanced edge detection, at a window level of -450 Hounsfield units (HU) and a window width of 1,350 HU. These settings have been shown to provide accurate measurement of luminal size as small as 0.5 mm in diameter [15], [16]. For repeated airway measurements in a given dog within each experimental protocol, adjacent anatomic landmarks, such as airway or vascular branching points, were used to locate and measure the airway area at the same anatomic cross sections. Representative images used for this quantitative analysis are shown later in the second Results panel.

Airway area on the HRCT images was analyzed using the airway analysis module of the Volumetric Image and Display Analysis (VIDA) image analysis software package (Dept. of Radiology, Division of Physiologic Imaging, Univ. of Iowa, Iowa City, IA) as previously described and validated [14], [17]. The HRCT images were transferred to a UNIX-based Sun workstation. An initial isocontour was drawn within each airway lumen, and the software program then automatically located the perimeter of the airway lumen by sending out rays in a spoke-wheel fashion to a predesignated pixel intensity level that defines the luminal edge of the airway wall. From the airway area (A) thus determined, mean airway diameter was calculated as 2(A/π)^1/2^. Intra- and inter-observer accuracy and variability of the software program using this HRCT technique in phantoms, consisting of rigid tubes to measure known areas, has been previously shown by us [15] and by others [17] to be highly resistant to operator bias. After segmentation of the parenchyma from the chest wall and mediastinum using a semi-automated process, lung volume was calculated by summing the volume of individual voxel elements contained within the region of interest using the Pulmonary Workstation 2 software (VIDA diagnostics, Iowa City, IA).

**Data Analysis:.** To compare the airway sizes at baseline and after relaxation with atropine, we analyzed the data using a standard least squares model with airway diameter as a percent of maximum as the outcome variable and airway pressure, the condition (baseline or after atropine), and lung volume as a percentage of maximum as the independent variables. Also, the Tukey HSD correction for post hoc multiple comparisons was used. To compare the airway size after histamine challenge by the aerosol route to the airway size after the catheter route, we analyzed the data using a standard least squares model with airway diameter as a percent of maximum as the outcome variable and airway pressure, the route of challenge (aerosol or catheter), and lung volume as a percentage of maximum as the independent variables again with the above Tukey HSD correction. To compare the lung volumes after histamine challenge by the aerosol route to the catheter route, we used one-way ANOVA. In addition, we also analyzed the change in transpulmonary pressure using a standard least squares model with the transpulmonary pressure as the outcome variable and lung volume as a percentage of maximum, pressure, and the 4 conditions as the independent variables. Significant difference were assumed if the p-value was <0.05.

## Results

**Figure 1:**
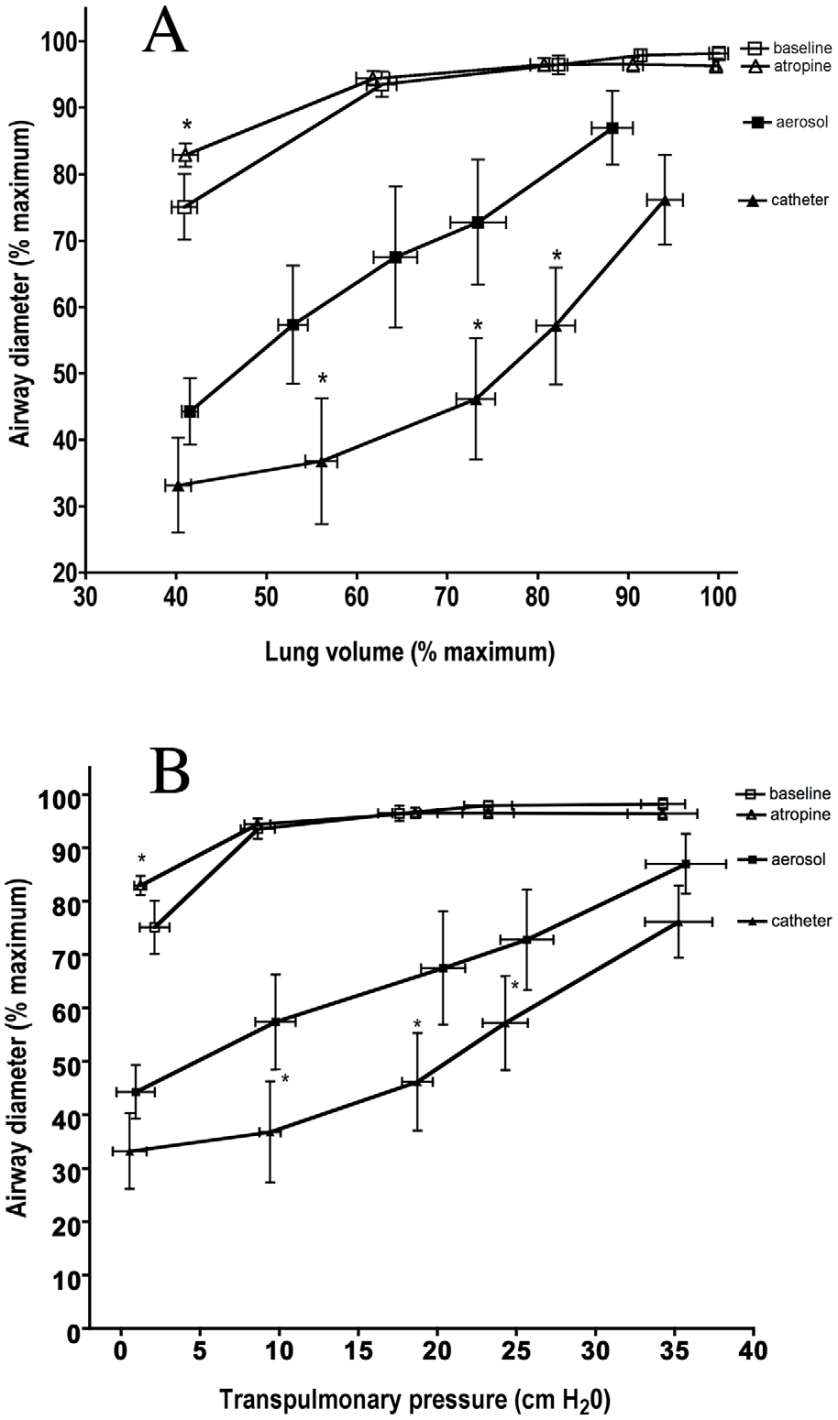
**A:** The mean (±SEM) airway diameter (as a percentage of the maximum airway diameter) vs. the mean (±SEM) lung volume (as a percentage of the maximum lung volume) at each airway pressure (0, 12, 25, 32, and 45 cm H_2_O) under the four conditions: baseline (before aerosol, open squares), atropine (before catheter, open triangles), aerosol histamine challenge (black squares), and individual airway catheter challenge (black triangles). **B:** The mean (±SEM) airway diameter (as a percentage of the maximum airway diameter) vs. the mean airway pressure under the same four conditions. There were significant differences in airway diameter and lung volume between the aerosol and catheter challenges. At every airway pressure above 0 cm H_2_O the airways were larger after aerosol challenge than after the local challenge (see text). Also, the aerosol challenge caused a greater stiffening of the lung. (*) At every airway pressure above 0 cm H_2_O, the lung volumes were larger after the catheter challenge than after the aerosol challenge (see text for p-values).

In each dog, the same individual airways were matched and measured on the separate days with the different challenges. In four dogs, we were able to match six airways, and in the other two dogs we matched five airways. These 3^rd^-4^th^ generation airways ranged in size from 3.9 to 10.5 mm in diameter at baseline and were in both the right and left lungs. In order to compare results from these different sized airways, individual airway diameter was normalized to a maximum defined as the diameter of the airway after smooth muscle relaxation at 45 cm H_2_O airway pressure. At baseline or when relaxed with atropine, the airways behaved similarly to increased lung volume or inflation pressure (Figures 1A and 1B), with no significant differences in mean airway diameter at any lung volume or airway pressure above functional residual capacity (FRC) (12 cm H_2_O, 25 cm H_2_O, 32 cm H_2_O and 45 cm H_2_O (P>0.05). At FRC, there was a significant difference between the airway size at baseline (75.0%±5%, mean±SEM) compared to after relaxation 83.0%±2%, P<0.05), indicating the presence of baseline tone.

With increased smooth muscle tone caused by whole lung histamine aerosol challenge, the mean airway size at FRC was 43.6%±5%. For the local direct airway challenge, the mean airway diameter at FRC was 32.0%±7% after challenge, but this was not significantly different from the airway size after aerosol challenge at FRC (P>0.05, **Figure 1A**). We also analyzed the data using a paired analysis (t-test) on the airway size (% maximum) at FRC by dog (n = 6), and this analysis also showed no statistical difference (p = 0.08).

**Figure 2:**
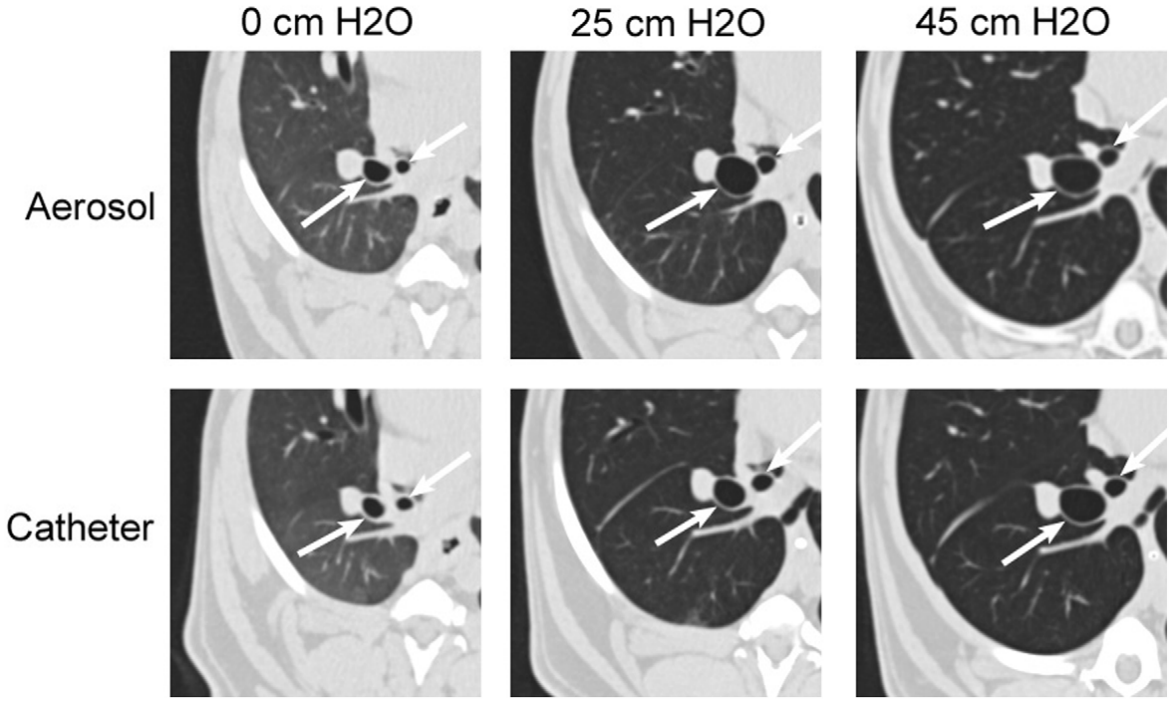
Representative HRCT images at three pressures with either the local catheter or whole lung aerosol challenge. Two individual airways identified by the arrows are seen in all the images. The airway sizes at 0 and 45 cm H_2_O appear similar with either challenge, but at 25 cm H_2_O, the airways with the catheter stimulation are clearly smaller than with the aerosol challenge.

Although the airways started out with the same degree of narrowing regardless of the method of stimulation, there were significant differences in how they responded to increasing inflation pressure, depending on the how the histamine was delivered. At airway pressures of 12, 25, and 32 cm H_2_O, the airways were larger after aerosol challenge than after the local challenge (P<0.05). At 45 cm H_2_O, again there was no difference in the airway size (P>0.05, **Figure 1B**). In addition, at 45 cm H_2_O, mean airway diameter was only 87.0%±6% and 76.1%±7% of maximum for the aerosol and locally challenged airways, respectively, indicating that even at the maximal inflation airways were still not fully dilated with either challenge. **Figure 2** shows representative HRCT images from one animal at three pressures with either the local catheter or whole lung aerosol challenge. Two airways identified by the arrows are seen in all the images. These qualitative images show a similar size at the lowest and highest pressures, but a clearly smaller size with the catheter stimulation at the midrange pressure as quantified in Figure 1.

Lung volume at FRC (0 cm H_2_O) was not different among the four conditions (P>0.05). At baseline or after atropine, lung volume expanded to a similar extent (P>0.05) at each airway pressure. However, when airway smooth muscle tone was increased by histamine, the lung volume expanded a smaller amount for each increase in airway pressure compared to the condition without histamine (P<0.05). There were also significant differences in lung volume between the aerosol and catheter challenges. As might be expected, the global aerosol challenge caused a greater stiffening of the lung. At 25 cm H_2_O (P = 0.02) and 32 cm H_2_O (P = 0.04) this stiffer lung resulted in volumes that were significantly smaller after the aerosol challenge than after the catheter challenge. At 12 cm H_2_O and 45 cm H_2_O, however, the difference in lung volume was not significant.

**Figure 3:**
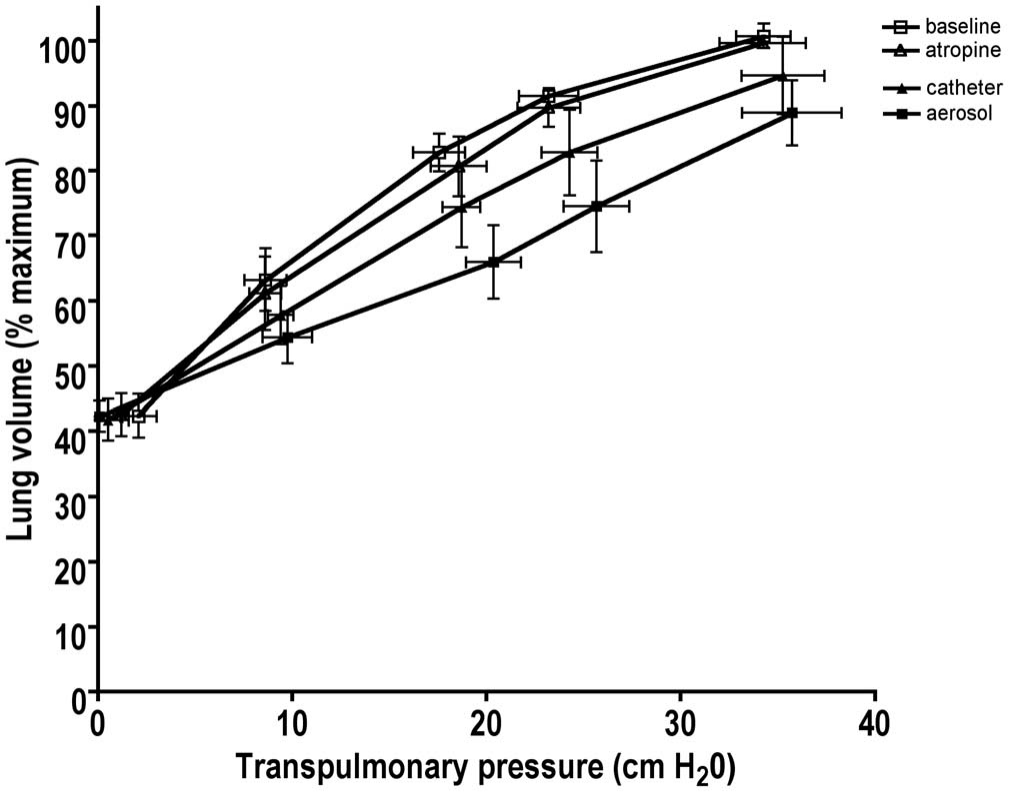
The mean (±SEM) lung volume (as a percentage of the maximum lung volume) and transpulmonary pressure (cm H_2_O) at each airway pressure (0, 12, 25, 32, and 45 cm H_2_O) under the four conditions: baseline (before aerosol, open squares), atropine (before catheter, open triangles), aerosol histamine challenge (black squares), and individual airway catheter challenge (black triangles) among four dogs. Transpulmonary pressure increased significantly with airway pressure (p<0.0001) in all four states, and there were no differences in transpulmonary pressures among the four states at any fixed level of airway pressure (p = 0.38).

**Figure 3** shows the mean lung volume as a function of transpulmonary pressure in the four dogs where we measured esophageal pressure. As expected, transpulmonary pressure increased significantly with airway pressure (p<0.0001) in all four states, and there were no differences in transpulmonary pressures among the four states at any fixed level of airway pressure (p = 0.21).

**Figure 4:**
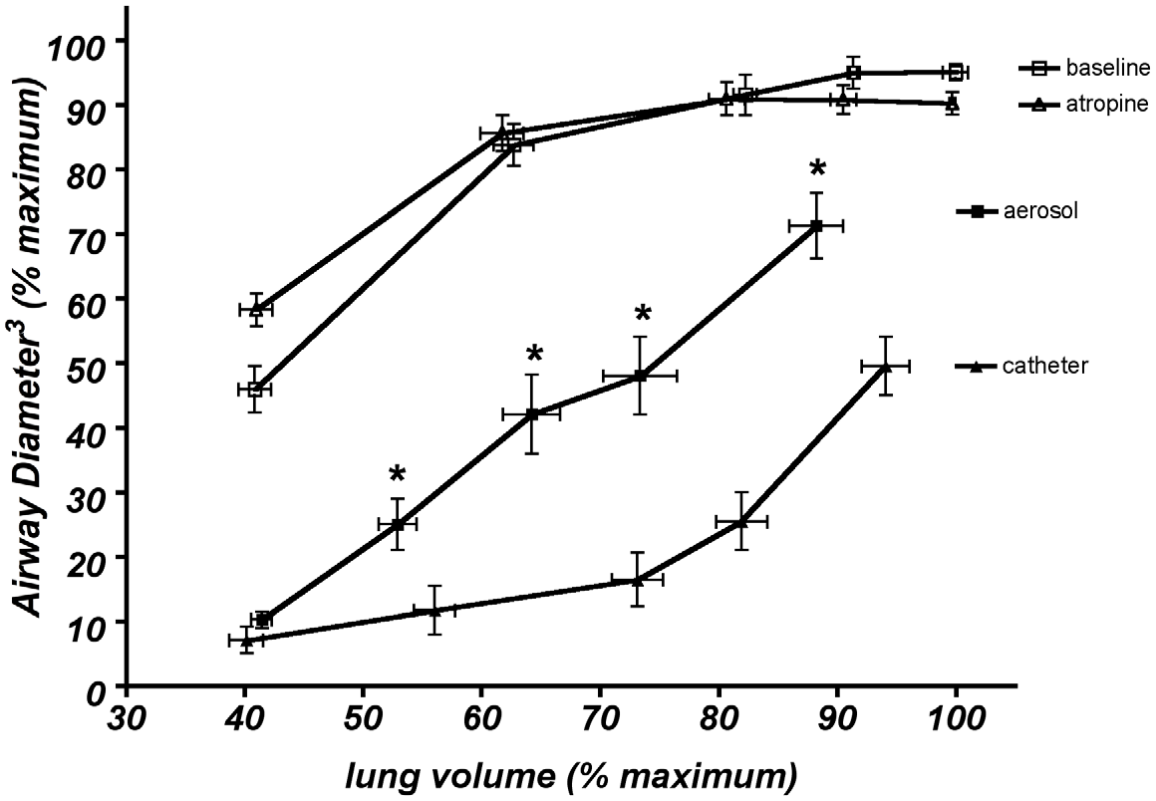
The relation between mean (±SEM) lung volume (as a percentage of the maximum lung volume) and mean (±SEM) airway diameter cubed. Both variables were measured at the five airway pressures (0, 12, 25, 32, and 45 cm H_2_O) under the four conditions: baseline (before aerosol, open squares), atropine (before catheter, open triangles), aerosol histamine challenge (black squares), and individual airway catheter challenge (black triangles). Airway diameter cubed is substantially and significantly less dependent on lung volume with the local catheter challenge significantly with airway pressure (p<0.001) at all lung volumes above FRC (0 cm H_2_0 Paw).

**Figure 4** shows the cube of the mean airway diameter vs. the mean lung volume. This graph scales the airway size to the same dimensions as the lung volume. After histamine challenge, at all lung volumes above FRC, this scaled airway was significantly greater (p<0.001) with the aerosol challenge than with the local challenge.

## Discussion

Parenchymal tethering produces a distending stress on the outer surface of airways, and with lung inflation, that stress increases. If lung volume is constant, any stiffening of the parenchyma should therefore lead to an increase in this distending stress on the airways relative to the baseline state with no exogenous contraction. The results of this study show that such parenchymal stiffening caused caused by generalized contraction of the entire airway tree does indeed have a protective effect on airway narrowing. When the parenchyma was stiffened by whole lung aerosol histamine challenge, at every lung volume above FRC, the airways were larger than when they were directly challenged with histamine to a similar initial constriction. These results are not a result simply of an increased lung volume, since at every increase in lung inflation above FRC, the lung volumes were significantly smaller with the global aerosol histamine compared to the local histamine challenge. If we had tried to compare airway diameters at matched lung volumes, then the airway size with the whole lung challenge would have been even greater.

Before continuing further discussion of possible mechanisms and implications, it is important to consider why the parenchyma stiffens when a smooth muscle agonist is administered. If we consider the parenchyma to mean the alveoli and alveolar walls, then there is little there that could result in a stiffer lung after histamine challenge. While contractile interstitial (Kapanci) cells have been described in the lung [18], these are generally only located around small blood vessels and very unlikely to have any effect on lung elasticity. Increased tone in the pulmonary blood vessels could have an effect on lung stiffness [19], but vascular smooth muscle does not contract with histamine. So, any increased lung stiffness must result entirely from contraction of airway smooth muscle, which exists in all airways down to the acini [20]. It is not known how much of the change in lung stiffness results from large or small airway constriction, but it is known that restricting the contractile agonist to just the conducting airways supplied by the bronchial circulation can cause a significant increase in lung elasticity [21]. In addition to stiffening the lung, smooth muscle contraction also results in increased tissue resistance [22]. Thus, when a whole lung agonist challenge is administered, not only are the airways narrowed, but there is also a stiffer, more viscous lung tissue [23]. These considerations highlight the fact that the airway tree cannot normally be separated from the parenchyma, since acute changes in parenchymal elasticity depend entirely on changes in airway smooth muscle tone, an idea that has been around for almost 100 years [24]. Our present results as shown in Figure 1B demonstrate for the first time that the converse is also true, i.e., that a stiffer parenchyma makes the airways stiffer as well. This effect is greatest at lung volumes close to FRC, and it progressively diminishes as lung volume approaches TLC.

Such a stiffened parenchyma as occurs with whole lung aerosol challenge acts as an increased elastic load on the airways that reduces the degree of narrowing. In contrast, when just a few airways are challenged directly through the catheter, without contraction of the rest of the airway tree, the parenchyma does not provide this increased load to attenuate the airway narrowing *in vivo*. These results support the concept that a stiffer lung tissue acts as a protective mechanism to minimize airway narrowing. We also observed that at the maximum lung inflation, the size of the airways after the aerosol challenge compared to the catheter challenge were not different (Figures 1A and B). This observation suggests that this protective effect on airway narrowing is limited, and that it can be overcome if the lung is inflated to sufficiently high volume.

Our conclusions, however, do not agree with several published studies [25], [26], [27]. Okazawa, et al concluded that in an excised rabbit lung the tethering effect of the lung parenchyma was not significantly increased by airway smooth muscle contraction. In interpreting their data, they used a continuum mechanics model of this interaction [28], where the bulk elastic modulus reflects the distensibility of the lung and the shear modulus (μ) reflects the ability of the lung to distort. The larger the shear modulus the smaller would be the change in airway diameter, and since μ is directly proportional to Ptp [29], an increased lung volume would also lessen the airway diameter changes. Okazawa et al [25] found that in a rabbit lung, carbachol had minimal effect on μ, with a small increase only at a Ptp of 12 cmH_2_O. We are not sure exactly how to account for this difference from our work, but we do note that our significant changes occurred at a Ptp range from ≈15 to 35 cmH_2_O, whereas Okazawa, et al made their measurements at a pressure range below 16 cmH_2_O. It is also unclear how these experimental studies fit with the theoretical modeling of Lambert and Paré [25], [26], [27], where it was concluded that the role of the parenchyma would only be effective at low lung volume.

In the study by Noble et al [26], porcine airway narrowing was examined in vivo with intact parenchyma, and in vitro with the parenchyma removed. They found no differences in airway narrowing at various transpulmonary pressures whether the parenchyma was intact or removed. In addition, there was no effect of cholinergic stimulation, and they thus concluded that parenchymal tethering is relatively unimportant in maintaining airway size. However, it is important to note that pig airways have a key structural difference from most other species, that being an unusually large amount of cartilage in their airwaywalls that can physically prevent severe narrowing [30]. This limitation was manifested in the study by Noble et al by the relaxed pig airway pressure-diameter curves, which were shown to be relatively flat and linear, and lacking the sharp knee at low pressure as seen in our Figure 1B.

There is, however, one potential explanation of our results that could be consistent with the weak parenchymal effects as found in these two studies. If one assumed that there was substantial airway closure with the aerosol challenge, then at the same total lung volume, a smaller fraction of the lung would be inflated and this would require a higher Ptp for this fraction. This higher Ptp would give rise to an increased shear and bulk modulus, and this would lead to increased airway size. We cannot rule this mechanism out, except to say that we have not seen any evidence of such closure in canine lung, at least down to the resolution of HRCT (≈1mm). If airway closure occurred in even smaller airways and was extensive enough over the whole lung, it could possibly account for our findings.

The results of this study also confirmed previous findings related to airway distension with lung inflation [14]. In that study, when the airways were either at baseline or relaxed, increasing airway pressure quickly led to a maximal size similar to what we showed in Figure 1B. This supports the notion that with minimal smooth muscle tone, canine airways are very distensible structures. When airway smooth muscle has increased tone, then the response to lung inflation depends on the degree of tone and the properties of the surrounding parenchyma. Increasing lung volume to a predicted lung capacity (i.e., at Ptp≈37 cmH_2_O) was not sufficient to fully dilate the airways, regardless of how the tone was generated. At TLC, mean airway diameters were only 87.0% and 76.1% of maximum for the aerosol and locally challenged airways respectively (Figure 1B). This was also the case in humans, where, with increased smooth muscle tone, airway size remains attenuated at TLC [14].

In comparing the two modes of challenge, there is one further difference that could possibly play a role. With the aerosol challenge, it was necessary to inflate the lung to deliver the aerosol, whereas the local challenge was delivered at FRC. It is known that both ventilation and deep inspiration can transiently decrease the airway responsiveness to an agonist challenge [1], [2], [3], [31], [32], [33], [34], [35], so it is possible that the decreased response to the aerosol challenge resulted from the delivery procedure. We attempted to minimize this potential effect by delivering the aerosol in only five breaths at a modest peak pressure of 15 cm H_2_O, which is only slightly above normal ventilation pressure at end inspiration. This is also much lower than the conventional Mch challenge procedure used to document hyperresponsiveness in asthmatic subjects, i.e., that of inhaling the agonist to TLC and holding one's breathe for 5 seconds. Our protocol here was in fact similar to the protocol for prevention of deep breaths to evaluate bronchoprotection and bronchodilation with just tidal volume breathing [1], [2], [3], [36]. In addition, we waited 3 minutes after each challenge, ventilating the dogs with the same normal tidal volume breaths as used after the local catheter challenge, before acquiring the first set of scans at FRC. We therefore feel that this mechanism was unlikely to be playing a major role in our observations.

The results in this study offer a possible explanation of a previously reported puzzling observation. We showed that with a high enough local dose of agonist, airways could be constricted to closure, and could not always be opened even at high lung volumes [12], [37]. This observation is at odds with many whole lung challenges where closure of large airways is not seen. Our present results offer a possible solution to this controversy, since without the parenchymal stiffening that occurs with whole lung challenge, it is easier for an individual airway to reach a smaller size even down to complete closure.

Finally, while induced bronchoconstriction by bronchial challenge is only a diagnostic and research tool for asthma, we offer a speculation about the potential clinical ramification of our observations. In clinical asthma, whether or not there is an inflammatory component, the final common pathway is airway smooth muscle contraction, and often enough the strength of this constriction may be greater than that which lung inflation can overcome. While not experimentally tested during an acute asthma attack, experimental studies with induced bronchoconstriction in asthmatic patients clearly show the inability of a deep inspiration to restore airway size [38], [39], [40], [41]. Thus, in clinical asthma, it is not simply increased airway smooth muscle contraction, but perhaps a lack of homogeneous parenchymal stiffening that contributes to the symptomatic airway hyperresponsiveness [23]. Heterogeneous airway narrowing could be detrimental in asthma, since such heterogeneity could mimic the effect we observed with the direct localized airway stimulation. In either case, decreased parenchymal tethering forces could account for the loss of bronchoprotection and bronchodilation seen in asthma and COPD with emphysema [1], [42].
